# Quantitative analysis of protease recognition by inhibitors in plasma using microscale thermophoresis

**DOI:** 10.1038/srep35413

**Published:** 2016-10-14

**Authors:** T. Dau, E. V. Edeleva, S. A. I. Seidel, R. A. Stockley, D. Braun, D. E. Jenne

**Affiliations:** 1 Comprehensive Pneumology Center, Institute of Lung Biology and Disease (iLBD), University Hospital, Ludwig Maximilians University and Helmholtz Zentrum München, Member of the German Center for Lung Research (DZL), Max-Lebsche-Platz 31, 81377 Munich, Germany; 2 Systems Biophysics and Functional Nanosystems, Ludwig Maximilians University München, Amalienstrasse 54, 80799 Munich, Germany; 3 Graduate School of Quantitative Biosciences, Ludwig Maximilans University, Feodor-Lynen-Str. 25, 81337 Munich, Germany; 4 Lung Investigation Unit, Queen Elizabeth Hospital Birmingham, Mindelsohn way, Edgbaston, Birmingham B15 2WB, UK; 5 Max-Planck-Institute of Neurobiology, Am Klopferspitz 18, D-82152 Martinsried, Germany

## Abstract

High abundance proteins like protease inhibitors of plasma display a multitude of interactions in natural environments. Quantitative analysis of such interactions *in vivo* is essential to study diseases, but have not been forthcoming, as most methods cannot be directly applied in a complex biological environment. Here, we report a quantitative microscale thermophoresis assay capable of deciphering functional deviations from *in vitro* inhibition data by combining concentration and affinity measurements. We obtained stable measurement signals for the substrate-like interaction of the disease relevant inhibitor α-1-antitrypsin (AAT) Z-variant with catalytically inactive elastase. The signal differentiates between healthy and sick AAT-deficient individuals suggesting that affinity between AAT and elastase is strongly modulated by so-far overlooked additional binding partners from the plasma.

One of the fundamental regulatory mechanisms in cell biology is the finely tuned interaction between enzymes and their substrates^1^. Binding between an enzyme and a substrate is most likely strongly influenced by the composition of the biological buffer system, for example by various plasma components^2^. However, functional assessment of protein-protein interactions in native environments has been a major challenge to date^3^,^4^.

Immunoassays and surface plasmon resonance are the current standard approaches to quantitate concentrations in plasma and to determine affinities of potential known targets^5^,^6^. However, both methods require surface immobilization of an antigen or an antibody, potentially imposing steric hindrance and molecular activity problems. Immobilization-free approaches based on fluorescence resonance energy transfer (FRET) require availability and labeling of both interacting partners^7^,^8^. Therefore, FRET-based methods cannot determine the affinity of a person-specific endogenous protein to a known target of interest in a plasma sample.

Microscale thermophoresis (MST) is a recently established immobilization-free affinity measurement technique that has been applied to characterize ligand-binder interactions and affinity constants ranging from pM to mM under challenging conditions^9^,^10^,^11^. MST employs the physical phenomenon of thermophoresis – movement of molecules in a temperature gradient. Biomolecules tend to move against the temperature gradient causing the depletion of a biomolecule in the heated spot. In the MST binding measurement, the depletion of the fluorescently labeled binder changes upon ligand binding. Measurement of depletion at increasing ligand concentrations results in a binding curve, which can be used to derive the affinity.

In our previous work using an MST-based approach, we showed how the affinity as well as the concentration of the ligand can potentially be determined in complex matrices such as blood serum^12^. The first limitation of this method is the sensitivity as only an additionally spiked but not initially present ligand could be detected in serum. Secondly, low affinity interactions remain a challenge, as they often require unrealizable high concentrations of the unlabeled binder to establish a complete binding curve.

An important clinically relevant example of protein interactions in plasma is the inhibition of the enzyme neutrophil elastase (NE) by α-1-antitrypsin (AAT). Low plasma levels of AAT predispose to emphysema and chronic obstructive pulmonary disease^13^,^14^. Decreased AAT levels are often caused by the relatively common Z-mutation (Glu342Lys)^15^ which accounts for 95% of patients with clinically relevant manifestations. Although all individuals with the Z-mutation have reduced AAT levels, development of lung emphysema is highly variable among ZZ carriers and some are minimally affected^13^,^14^,^16^.

The interaction between NE and AAT has been well studied *in vitro* using the two purified interacting proteins in standard buffers^17^. AAT inhibits NE irreversibly in a two-step reaction^18^. The first and rate-determining step is the formation of a reversible encounter complex between NE and AAT, where AAT mimics a substrate^19^,^20^. Previous approaches focused only on the concentration of AAT^5^,^13^,^15^,^16^, although kinetically efficient formation of this encounter complex also depends on the dissociation constant between NE and AAT^19^,^20^.

In this work, we developed a novel assay that overcomes the limitations of the previous approaches and allowed us to characterize the formation of the NE-AAT encounter complex at the given AAT level of a blood plasma sample under constant equilibrium conditions.

## Results

### Method development: combining concentration and affinity

**Figure 1:**
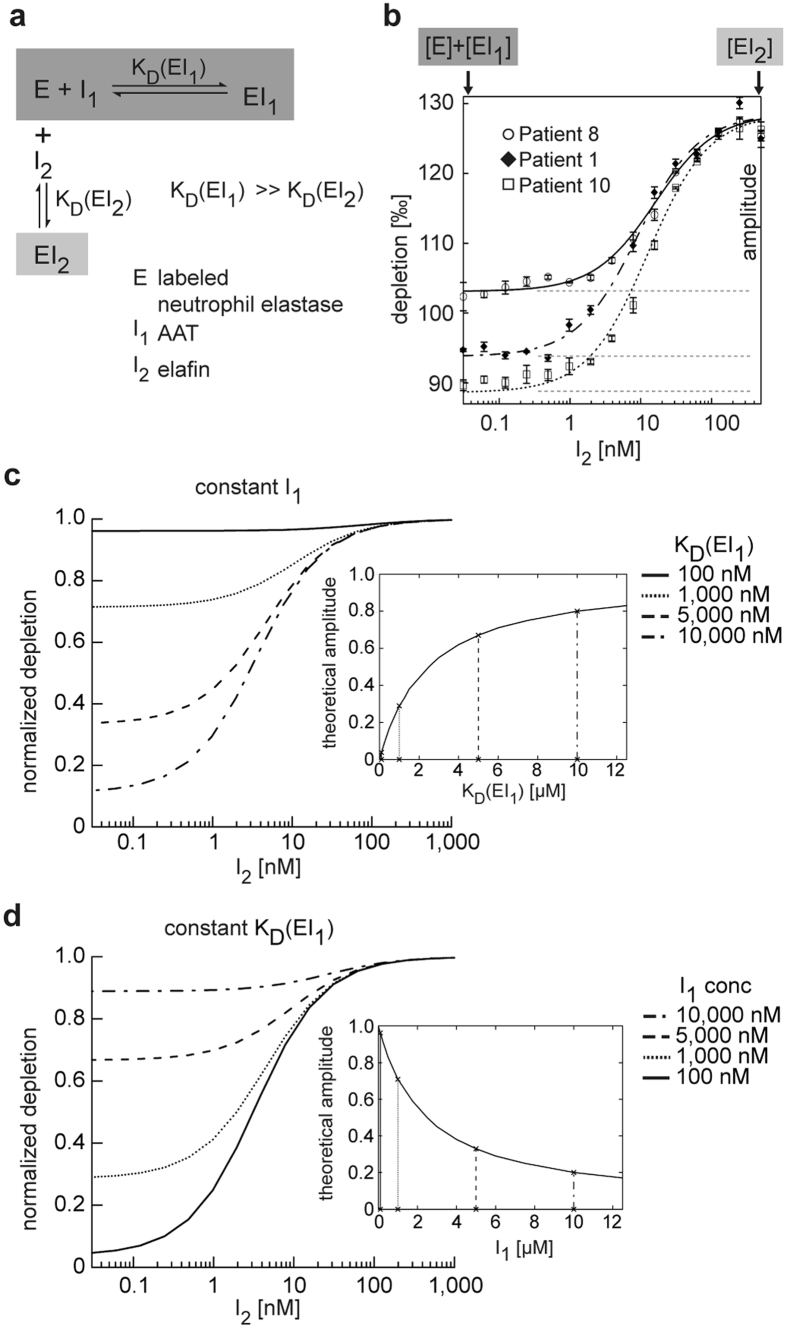
Dependency of the amplitude on the AAT concentration and the dissociation constant of NE-AAT binding. (**a**) Describes the theoretical background of the method. At each data point, the inhibitor I_1_ (here: AAT) competes with I_2_ (here: elafin) for the enzyme E (here: labeled inactive NE). The affinity between E and I_2_ is much higher than the affinity between E and I_1_. (**b**) To a constant amount of plasma (7.5%) containing I_1_ and a constant amount of E (500 pM) increasing concentrations of I_2_ were added and the thermophoretic depletion measured. Titration curves with theoretical fits are given for three individuals (patients 1, 8 and 10) as examples in (**b**). All individuals were homozygous for the Z-variant AAT. Each depletion value is given as mean ± S.D. from three technical replicates. The amplitude is defined as the difference of minimal depletion signals in the low end range (dark grey box with arrow) and maximal depletion signals in the high end range of I_2_ concentrations (light grey box with arrow). The thermophoretic depletion of free E is smaller than that of E bound to I_1_ or I_2_, and depletion at each I_2_ concentration indicates the proportion of bound E. (**c,d**) Are simulations according to the mass action laws. (**c**) Shows that the theoretical amplitude (simulated as the amount of bound E) increases when the dissociation constant between E and I_1_ increases. (**d**) On the other hand, the theoretical amplitude decreases with the increasing concentrations of I_1_.

In our assay, a low affinity inhibitor I_1_ (here: AAT) competes with a high affinity inhibitor I_2_ (here: elafin) for a catalytically inactive labeled enzyme E (here: NE) (Fig. 1a). Inactivation of E was important to prevent the removal of I_1_ from the equilibrium by the irreversible formation of a covalent complex, thereby allowing us to analyze the encounter reaction in the plasma environment.

The labeled E at a fixed final concentration of 500 pM was mixed with 12-fold diluted plasma containing the analyte I_1_. To obtain binding curves, increasing amounts of I_2_ were then added and thermophoretic depletion of free E and E bound to I_1_ (EI_1_) or I_2_ (EI_2_) was measured for each concentration of I_2_ (Fig. 1a,b). We observed that the depletion in the low end range of I_2_ concentrations varied between plasma samples while the depletion in the high end range of I_2_ concentrations did not change (Fig. 1b).

To compare the binding curves, we introduced a normalized parameter, the thermophoretic amplitude, by calculating the difference between the depletion signals in these two I_2_ concentration ranges (Fig. 1b, Supplementary Information 1). We observed that the thermophoretic amplitude varied between plasma samples. We hypothesized that I_1_ concentrations and, more importantly, I_1_ affinities to E are not necessarily constant in the person-specific proteomic environment of plasma samples (Fig. 1b).

To characterize the system at different affinities between I_1_ and E (Fig. 1c) and different I_1_ concentrations (Fig. 1d), we described it with the mass action law equations and simulated the corresponding binding curves. The simulation results indicate that the thermophoretic amplitude increases with an increasing K_D_(EI_1_) (Fig. 1c small insertion graph) and decreases with increasing I_1_ concentrations (Fig. 1d small insertion graph). Hence, the thermophoretic amplitude reports on both I_1_ concentration and K_D_(EI_1_) (a detailed mathematical analysis is provided in the Supplementary Information 2).

### Thermophoretic amplitude is better correlated with disease status

To evaluate whether the thermophoretic amplitude that includes affinity variations represents a better lung function parameter than AAT concentration alone, we determined both parameters in plasma samples of individuals homozygous for the AAT Z-variant. We then compared these to FEV_1_ (the forced expiratory volume in one second) expressed as the percentage (%) predicted for age, sex, height, and race, which is widely used to assess the level of airway obstruction and bronchoconstriction in AAT-deficient individuals^16^,^21^.

**Figure 2:**
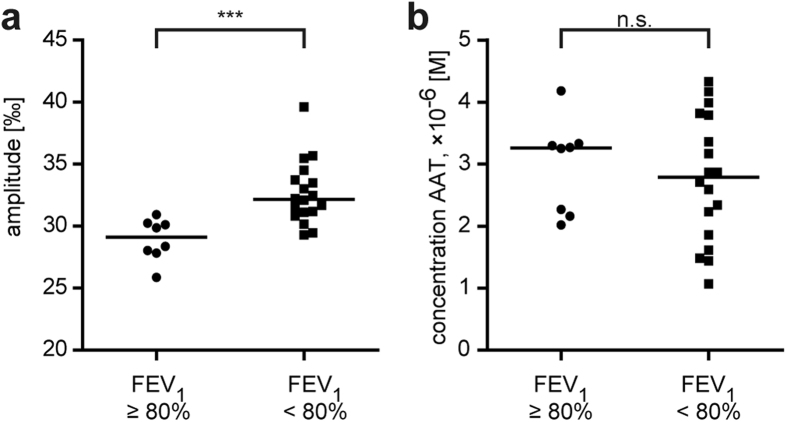
Thermophoretic amplitude, but not plasma concentration of AAT is associated with disease severity. (**a**) ZZ-carriers (n = 26) were divided into two groups with high FEV_1_ (≥80%, n = 8) and low FEV_1_ (<80%, n = 18), which is a clinical parameter of airway obstruction. The thermophoretic amplitude was significantly lower (P = 0.0008) in samples with high FEV_1_ (***P < 0.001; ns not significant). The amplitude is given as a mean value determined from at least two independent experiments. (**b**) The concentration of functional AAT was not significantly different (P = 0.6368) between the groups with high FEV_1_ (≥80%) and low FEV_1_ (<80%). The AAT concentration was measured twice by elastase titration in duplicates, and is given as a mean value here.

In order to avoid measuring the concentration of inactive AAT, we titrated the concentration of functional plasma AAT with purified and active site-titrated NE. We observed a significantly lower thermophoretic amplitude (P = 0.0008) with plasma samples from individuals showing a high FEV_1_ (≥80%) than from those with low FEV_1_ (<80%) (Fig. 2a), while the concentration of functional AAT did not differ between the two groups (P = 0.6368). Hence, inclusion of affinity with our method resulted in a much better relationship to disease status and severity. To explain the discrepancy between the two quantification methods, we reasoned that K_D_(EI_1_) of the Z-variant in fact varied in the plasma samples.

### Z-variant AAT is sensitive to plasma environments

**Figure 3:**
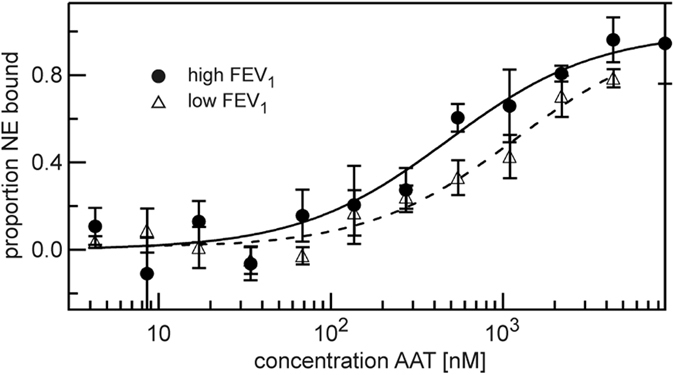
Plasma-dependent change in affinity of NE-AAT binding. We compared the affinity between AAT and NE in two pools of plasma from individuals with high FEV_1_ (≥80%, n = 8) and low FEV_1_ (≤50%, n = 12). The affinity between Z-variant of AAT and NE was higher in plasma with high FEV_1_ (≥80%) (K_D_(EI_1_) = 500 ± 100 nM) compared to low FEV_1_ (≤50%) (K_D_(EI_1_) = 1300 ± 250 nM). Presented binding curves represent example measurements where each measurement point (mean ± S.D.) was derived from three technical replicates. Fitted binding curves and K_D_(EI_1_) values (mean ± S.D.) were derived from global fitting of four measurements (three independent protein expressions). The measurements were performed in 7.5% plasma and with 5 nM of labeled NE.

To investigate whether affinity is indeed sensitive to a given plasma environment, we determined the K_D_(EI_1_) of the AAT Z-variant in the two AAT-deficient plasma pools from persons with high (≥80%) and low FEV_1_ (≤50%) values (Fig. 3). To facilitate the recombinant production of the aggregation-prone Z-variant, we combined three stabilizing mutations derived from the work of Lee *et al*.^22^ – Met374Ile, Ser381Ala, Lys387Arg – and introduced them into the Z-variant. As a control, we expressed AAT without the Z-mutation but containing the same stabilizing mutations. The affinity of the Z-variant to NE was more than two times better in the plasma pool from individuals with high FEV_1_ (≥80%) (K_D_(EI_1_) = 500 ± 100 nM) than from individuals with low FEV_1_ (≤50%) (K_D_(EI_1_) = 1300 ± 250 nM), confirming the plasma-dependent change in the affinity between NE and Z-variant AAT as observed with our newly developed assay. Additionally, we found that the Z-mutation improves the encounter reaction of AAT with NE (Supplementary Fig. 1a) and Z-variant AAT is more susceptible to plasma components than control AAT (Supplementary Fig. 1b).

## Discussion

By incorporating a high affinity ligand in thermophoretic measurements, we expanded the spectrum of applications towards the low affinity range where e.g. enzyme-substrate interactions take place. This new approach allowed us to detect unexpected plasma-dependent interferences with AAT, suggesting that the functional capacity of the Z-variant AAT is determined by the heterogeneity of other plasma components, which modulate the affinity of AAT towards target enzymes. For example, it was reported^23^,^24^ and has been confirmed by our group (unpublished) that lipoproteins bind to wildtype AAT, thereby influencing its inhibition of NE and thus could also affect the reactivity of the Z-variant in the plasma environment.

The analysis of the interaction between AAT and NE in plasma with our assay provides a possible explanation of the variability in disease development in ZZ carriers. Normally, the reduced AAT plasma levels in ZZ carriers are compensated by the enhanced affinity of Z-variant AAT to NE. However, the enhanced affinity between Z-variant AAT and NE can be disrupted by plasma factors, especially in individuals who have higher concentrations of interfering plasma components.

Our work underscores the importance of the endogenous personally unique and distinct environment where substrates interact with enzymes with relatively low affinity in a complex web of other proteins and lipid components. Such personalized variations and effects have often been overlooked. Our approach may open a way to study how other components from the native environments interfere with biochemical reactions.

## Methods

### Production of recombinant proteins

As the natural Z-variant is on a M1(Ala213) background, we amplified a cDNA fragment encoding the M1(Ala213) variant using the primer DJ3689 (5′-GACTTCCACGTGGACCAGG**C**GACCACCGTGAA-3′) and DJ3613 (5′-GATG*ACCGGT*TTTTTGGGTGGGATTCACCACTT-3′) (*cursive*: restriction site; **bold**: mutation) and cloned it into the *Pml* I and *Age* I site of the previously described M1(Val213) AAT construct in pTT5 plasmid (Perera *et al*.^25^. To introduce the Z-mutation (Glu342Lys), a PCR fragment was generated with the primers DJ3557 (5′-CCAC*GATATC*ATCACCAAGTTCCT-3′) and DJ3558 (5′-GTATGG*CCTCGAGG*AACATGGCCCCAGCAGCTTCAGTCCCTTTCTTGTCGATGGT-3′) and inserted into the M1(Ala213) AAT pTT5 plasmid between the *Eco* RV and *Abs* I sites. The three stabilizing mutations (M374I, S381A, K387R) were introduced by PCR using the following forward and backward primers DJ 3646 (5′- CAC*GATATC*ATCACCAAGTTCCTGGAAAATGAAGACAGAAGGTCTGCCGACTTACATTTACC-3′) and DJ3696 (5′-ATG*ACCGGT*TTTTTGGGTGGGATTCACCACT**C**TTCCCATGAAGAGGGGAG**C** CTTGGTATTTTGTTCAAT**A**ATTAAG-3′) respectively. The product was inserted into the *Eco* RV and *Age* I sites.

To produce a catalytically inactive variant of NE, we mutated the Ser195 to Ala195 by inserting an oligo duplex (DJ3532 (5′-GTGAACGTATGCACTCTGGTGCCACGTCGGCAGGCAGGCATCTGCTTCGGGGACGCT-3′) and DJ3533 (5′-CGTCCCCGAAGCAGATGCCTGCCTGCCGACGTGGCACCAGAGTGCATACGTTCACAC-3′)) into the *Alf I* site of the previously described wildtype mouse NE construct in pTT5 (Dau *et al*.^26^. To enable site-specific labeling of NE, we added a C-terminal short peptide with a cysteine flanked by three aspartate residues on each side by the insertion of an oligoduplex (DJ3632 (5′- CTAGCGACGACGATTG CGACGATG ATC-3′) and DJ3633 (5′- CTAGGATCATCGTCGCAATCGTCGTCG-3′)) into the *Avr* II site.

All proteins were expressed in HEK293 EBNA cells (Yves Durocher, National Research Council Canada, Montreal, Canada) in FreeStyleTM 293 expression medium (Thermo Fisher Scientific Inc.), 1% Pluronic and G418 (25 μg ml^−1^) at 37 °C and 8% CO_2_.

### Labelling of NE

First, we added 1 mM DTT to the recombinant NE in storage buffer (20 mM Na_2_HPO_4_, 300 mM NaCl pH 7.4) and incubated it for two hours at room temperature to reduce all cysteine-tags. Then we removed DTT by precipitating NE with 75% ammonium sulfate. This reduction and precipitation step was repeated once. NE was dissolved in storage buffer and incubated at room temperature for one hour after adding a 5-fold molar excess of dye (Alexa Fluor^®^ 647 NHS Ester, ThermoFisher Scientific). To remove the excess dye, the solution was added to PD MiniTrap G-10 column (GE Healthcare) according to the manufacturers instruction.

### Determination of AAT concentration in blood plasma

After titrating trypsin (Sigma-Aldrich, St. Louis) with 4-nitrophenyl 4-guanidinobenzoate (Sigma-Aldrich, St Louis, USA) in veronal buffer, AAT (Athens Research & Technology, Athens, GA, USA) was titrated against trypsin. A dilution series of AAT was incubated with a constant amount of trypsin at 37 °C for 1 hour. The residual activity was measured using Boc-Gln-GLy-Arg-AMC. Thereafter, a dilution series of the titrated AAT was incubated with a constant volume of active human NE (Elastin Products Company, Inc., Owensville) at 37 °C for 1 hour. The remaining activity was measured with MCA-GEAIPTSIPPEVK(Dnp)-rr (EMC microcollections, Tübingen, Germany). All reactions were performed in 150 mM NaCl, 50 mM Tris, 0.01% Triton-X-100, pH 7.4.

Active site titrated neutrophil elastase was added to a dilution series of plasma and incubated at 37 °C for 1 hour. The plasma was diluted in 150 mM NaCl, 50 mM Tris, 0.01% Triton-X-100, pH 7.4. The residual activity was measured using MCA-GEAIPTSIPPEVK(Dnp)-rr (EMC microcollections, Tübingen, Germany).

### Determination of the concentration of recombinant AAT

A dilution series of AAT-variants was incubated with 1.6 nM active site titrated human NE at 37 °C for 1 hour. The residual activity was measured using MCA-GEAIPTSIPPEVK(Dnp)-rr (EMC microcollections, Tübingen, Germany).

### Statistical analysis

Results are given as median values. A Mann-Whitney test without assuming Gaussian distribution was applied to all studies (***P < 0.001). Statistical analyses were done with GraphPad Prism 6 software (GraphPad Software).

### Development of the Binding Model for Simulations

Mass action law equations with two dissociation constants were used to describe the dependency of free NE on the concentration of AAT and on the affinity of AAT to NE in the presence of two NE ligands – AAT and elafin.


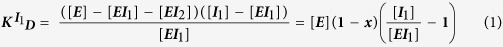



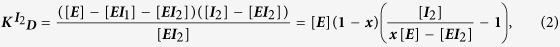


where


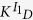
 – dissociation constant of NE – AAT interaction,


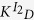
 – dissociation constant of NE – elafin interaction,

[E], [I_1_], [I_2_] – total concentrations of NE, AAT, and elafin in the reaction, respectively,

[EI_1_] – concentration of NE-AAT complex in the reaction,

[EI_2_] – concentration of NE-elafin complex in the reaction,


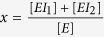
 – fraction of occupied NE,

Equation (2) was solved for [E], which was then applied to equation (1) to yield a cubic equation for *x.* The cubic equation was solved numerically in Igor Pro 5.03 and the smallest real root of the equation was then used to plot the fraction of bound NE *x* against different elafin concentrations I_2_ to simulate binding curves.

### Determination of the thermophoretic amplitude

Human recombinant elafin (Sigma-Aldrich) was serially diluted (1:1) over five orders of magnitude in PBS. Separately, plasma was diluted (final concentration: 7.5%) with storage buffer substituted with 0.02% Tween 20 and anti-photobleaching enzyme and substrate components (Monolith Anti Photobleach Kit, NanoTemper Technologies) were added. This plasma solution was mixed 1:1 with PBS for background measurements. After adding fluorescently labeled NE (final concentration: 500 pM) to the plasma solution, we mixed this 1:1 with each concentration of the serial elafin dilution. The samples were loaded into the Monolith™ NT.115 premium coated capillaries (NanoTemper Technologies), and incubated at 22 °C for 2 hours. The samples were measured in the instrument (Monolith NT.115Pico, NanoTemper Technologies) at 22 **°**C using 60% light-emitting diode and 40% infrared laser (IR) powers with IR laser on/off times of 25/5 seconds. Each dilution point was measured in triplicates. For each plasma sample, the whole procedure was repeated three times to yield independent triplicates.

In Matlab R2014a (8.3.0.532), the time trace of the background signal was subtracted from the time traces of the sample signals. Then, the fluorescence after the temperature jump and equilibrated thermophoresis was normalized to the fluorescence before the IR laser heating yielding fluorescence depletion values. Fluorescence depletion values of three technical replicates (mean ± S.D.) were plotted in per mille units (‰) on a linear y-axis against the concentration of the serially diluted elafin on the log_10_ x-axis resulting in binding curves. The binding curves were shifted so that the depletion value at the baseline, where elafin concentration is saturating, was the same. In Igor Pro 5.03, a weighted fit to the quadratic solution of the mass action law was performed to yield the amplitude *A* of each curve:


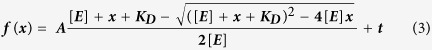


where

*K*_*D*_ – dissociation constant of NE – elafin interaction,

[E] – total concentration of NE,

*x* – total concentration of elafin,

*A* – the amplitude of the curve,

*t* – y-offset of the curve,

with *A*, *t*, and *K*_*D*_ as free fit parameters.

### Determination of dissociation constant between AAT and NE

Freshly purified AAT was serially diluted (1:1) over five orders of magnitude in storage buffer. Separately, plasma was diluted in storage buffer substituted with 0.2% Tween 20 (final concentration: 7.5%) and anti-bleaching reagents were added. This plasma solution was mixed 1:1 with storage buffer for background measurements. Fluorescently labeled NE (final concentration: 5 nM) was added to the plasma solution and mixed 1:1 with the serial dilution of AAT. The samples were incubated at 22 °C for 2 hours and measured at 22 °C in the instrument using 40% light-emitting diode and 40% infrared laser (IR) powers with IR laser on/off times of 20/5 seconds.

Binding curves were obtained as described for the determination of the thermophoretic amplitude. They were then additionally normalized to 0 at the lowest AAT concentration and to 1 at the highest AAT concentration. This corresponds to the proportion of bound NE at each titration point. The global fit of at least three replicates to the quadratic solution of the mass action law was performed to yield the dissociation constant of each curve.

### Plasma samples

FEV_1_ was determined post bronchodilator treatment and according to the British Thoracic Society/Association of Respiratory Technicians and Physiologists (BTS/ARTP) guidelines as described previously^21^. All subjects provided written informed consent, and ethical approval was obtained for all aspects of this study (South Birmingham Research Ethics Committee LREC 3359).

## Additional Information

**How to cite this article**: Dau, T. *et al*. Quantitative analysis of protease recognition by inhibitors in plasma using microscale thermophoresis. *Sci. Rep.* **6**, 35413; doi: 10.1038/srep35413 (2016).

## Supplementary Material

Supplementary Information
