# Supplementary material for: Quantitative analysis of protease recognition by inhibitors in plasma using microscale thermophoresis
Source: Sci Rep. 2016 Oct 14;6:35413. doi: 10.1038/srep35413 (PMC5064372; doi:10.1038/srep35413)
Supplement: Supplementary Information [file srep35413-s1.pdf]

Quantitative analysis of protease recognition by inhibitors in plasma using microscale thermophoresis

Dau T.*[1], Edeleva E.V.*[2,3], Seidel, S.A.I.[2], Stockley R.A.[4], Braun D.[2], Jenne D.E.[1,5]

*shared first author

Affiliations

[1]Comprehensive Pneumology Center, Institute of Lung Biology and Disease (iLBD), University Hospital, Ludwig Maximilians University and Helmholtz Zentrum München, Member of the German Center for Lung Research (DZL), Max-Lebsche-Platz 31, 81377 Munich, Germany

[2]Systems Biophysics and Functional Nanosystems, Ludwig Maximilians University München, Amalienstrasse 54, 80799 Munich, Germany

[3]Graduate School of Quantitative Biosciences, Ludwig Maximilans University, Feodor-Lynen-Str. 25, 81337 Munich, Germany

[4]Lung Investigation Unit, Queen Elizabeth Hospital Birmingham, Mindelsohn way, Edgbaston, Birmingham B15 2WB, UK

[5]Max-Planck-Institute of Neurobiology, Am Klopferspitz 18, D-82152 Martinsried, Germany

Supplement

Supplementary Information 1: Theoretical background

We defined the thermophoretic amplitude as the difference between minimal and maximal depletion signals in the low and high end range of I_2 concentrations, respectively (Fig.1b). To better illustrate how the thermophoretic amplitude behaves in these two concentration ranges, we have analyzed the theoretical equilibrium for both situations separately in more detail.

In the low end range of I_2 concentrations, interactions between I_2 and E are almost non-existent. Therefore, the proportion of bound E depends solely on its interaction with I_1 (Fig. 1b, dark grey box). The proportion of bound E at this point is a function of both, the concentration of I_1 as well as the dissociation constant between E and I_1 ($K_D(EI_1)$) in plasma. Higher concentrations of I_1 shift the equilibrium towards EI_1 complexes, while a higher $K_D(EI_1)$ results in a decrease of EI_1 complexes (Fig 1a, dark grey box). The thermophoretic depletion of I_1-bound E is considerably larger than that of the free E. Hence, the measured thermophoretic depletion reflects the proportion of E bound to I_1 in the mixture, which varies between the plasma samples.

At maximal concentration of I_2, all E is bound to I_2 independent of the other parameters due to the high affinity between E and I_2 (Fig. 1b, light grey box). The thermophoretic depletion is the depletion of EI_2 complexes and is the same for all the plasma samples and only depends on the amount of labeled probe E spiked into a sample. Thermophoretic depletion of I_2-bound E is also considerably larger than that of the free E.

Taken together, the thermophoretic amplitude depends on the proportion of I_1-bound E in the sample at I_2 concentrations approaching zero. We obtain the largest amplitude when all E is free at lowest I_2 concentration in equilibrium. Accordingly, the thermophoretic amplitude decreases with decreasing $K_D(EI_1)$ values and increasing I_1 concentrations (Fig. 1c, d, smaller insertion graphs).

Supplementary Information 2: Mathematical analysis of the system

Defining the system

Let us consider the chemical binding equilibrium between ligands I_1 and I_2 and fluorescently labeled binder E, where I_1 is AAT in plasma, I_2 is elafin, and E is labeled elastase in the system studied in this paper.

$$E + I_1 + I_2 \rightleftarrows EI_1 + EI_2$$

The binding is recorded as the difference in the thermophoretic depletion between molecule E in the free state and in the bound states EI_1 and EI_2.

Thermophoretic depletion of a molecule is described by its Soret coefficient $S_T^{molecule}$. We assume that Soret coefficients of free and bound species of molecule E in the sample are different:

$$S_T^E \neq S_T^{EI_1} \neq S_T^{EI_2}$$

Detected fluorescence

Steady-state concentration of molecule E, c^E, at the position where the temperature is increased by small ΔT (5-10 K) can be found as:

$$\frac{c^E}{c_0^E} = e^{-S_T^E \Delta T} \approx 1 - S_T^E \Delta T \qquad eq.(1)$$

where c_0^E is concentration of molecule E when $\Delta T = 0$ [1]. (Notice, that subscript "0" in the concentration term will always denote the cold concentration at $\Delta T = 0$).

Similarly for EI_1 and EI_2:

$$\frac{c^{EI_1}}{c_0^{EI_1}} = e^{-S_T^{EI_1} \Delta T} \approx 1 - S_T^{EI_1} \Delta T \qquad eq.(2)$$

$$\frac{c^{EI_2}}{c_0^{EI_2}} = e^{-S_T^{EI_2} \Delta T} \approx 1 - S_T^{EI_2} \Delta T \qquad eq.(3)$$

Fluorescence detected from a sample of E, EI_1, and EI_2 mixture can be expressed as sum of fluorescence detected from molecule E in free and bound states:

$$F = F_E + F_{EI_1} + F_{EI_2}$$

Detected fluorescence can be further expressed as a product of quantum efficiency of the dye attached to the molecule $f^{molecule}$ and concentration of a particular species:

$$F = f^E c^E + f^{EI_1} c^{EI_1} + f^{EI_2} c^{EI_2}$$

We focus on a likely case that quantum efficiencies of bound and unbound species are equal:

$$F = f c^E + f c^{EI_1} + f c^{EI_2} \qquad eq.(4)$$

From $eq.(1) - (4)$, fluorescence detected after the sample was heated by small ΔT can be expressed as:

$$F(\Delta T) = f c^E + f c^{EI_1} + f c^{EI_2} - \left(f c_0^E S_T^E \Delta T + f c_0^{EI_1} S_T^{EI_1} \Delta T + f c_0^{EI_2} S_T^{EI_2} \Delta T \right) \qquad eq.(5)$$

Let us introduce a fraction of molecule E bound to molecule I_1, P_1:

$$P_1 = \frac{c_0^{EI_1}}{c_0^{EI_1} + c_0^E} \qquad eq.(6)$$

Similarly, a fraction of molecule E bound to molecule I_2, P_2:

$$P_2 = \frac{c_0^{EI_2}}{c_0^{EI_2} + c_0^E} \qquad eq.(7)$$

Substituting $\square_0^{EI_1}$ and $c_0^{EI_2}$ in $eq.(5)$ with c_0^E, P_1, and P_2, fluorescence in the heated state can be expressed as:

$$\begin{aligned} F(\Delta T) = {} & fc_0^E + fc_0^E \frac{P_1}{1-P_1} + fc_0^E \frac{P_2}{1-P_2} \\ & - \left(fc_0^E S_T^E \Delta T + fc_0^E \frac{P_1}{1-P_1} S_T^{EI_1} \Delta T + fc_0^E \frac{P_2}{1-P_2} S_T^{EI_2} \Delta T \right) \qquad eq.(8) \end{aligned}$$

Thermophoretic depletion

Depletion by definition is the fraction of fluorescence detected in the heated state over the fluorescence detected in the cold state:

$$Depletion = \frac{F(\Delta T)}{F(\Delta T = 0)}$$

From $eq.(8)$ and then $eq.(6) - (7)$ after algebraic transformations follows that $Depletion$ is given by:

$$Depletion = 1 - \left(S_T^E \frac{c_0^E}{c_0^E + c_0^{EI_1} + c_0^{EI_2}} + S_T^{EI_1} \frac{c_0^{EI_1}}{c_0^E + c_0^{EI_1} + c_0^{EI_2}} + S_T^{EI_2} \frac{c_0^{EI_2}}{c_0^E + c_0^{EI_1} + c_0^{EI_2}} \right) \Delta T \qquad eq.(9)$$

Amplitude

We defined *Amplitude* in the paper as the difference between the thermophoretic depletion at negligible concentration of I_2 in the reaction and after adding the maximal concentration of I_2:

$$\begin{aligned} Amplitude &= Deplet\square on\ \left(at\ c_0^{I_2} \approx 0\right) - Depletion\ \left(at\ c_0^{I_2}\ such\ that\ c_0^{EI_1} = 0\ and\ c_0^E = 0\right) \\ &= Depletion_{start} - Depletion_{end} \qquad eq.(10) \end{aligned}$$

From $eq.(9)$ follows:

$$Depletion_{start} = 1 - \left(S_T^E \frac{c_0^E}{c_0^E + c_0^{EI_1}} + S_T^{EI_1} \frac{c_0^{EI_1}}{c_0^E + c_0^{EI_1}} \right) \Delta T \qquad eq.(11)$$

$$Depletion_{end} = 1 - S_T^{EI_2} \frac{c_0^{EI_2}}{c_0^E + c_0^{EI_1} + c_0^{EI_2}} \Delta T = 1 - S_T^{EI_2} \Delta T \qquad eq.(12)$$

From $eq.(10) - (12)$:

$$Amplitude = S_T^{EI_2}\Delta T - (S_T^E \frac{c_0^E}{c_0^E + c_0^{EI_1}} + S_T^{EI_1}\frac{c_0^{EI_1}}{c_0^E + c_0^{EI_1}})\Delta T \qquad eq.(13)$$

Let us analyze $eq.(13)$. Soret coefficients $S_T^{EI_2}$, S_T^E, and $S_T^{EI_1}$ are intrinsic properties of molecules EI_2, E, and EI_1 that define behavior of these molecules in the temperature gradient. $\frac{c_0^E}{c_0^E+c_0^{EI_1}}$ is the proportion of free E at equilibrium when only E and I_1 are present in the sample. Similarly, $\frac{c_0^{EI_1}}{c_0^E+c_0^{EI_1}}$ is the proportion of complex EI_1 at equilibrium when only E and I_1 are present in the sample. Thus, the amplitude of binding curves that we obtain in our measurements is defined as the difference between the Soret coefficient of the complex EI_2 and the sum of products of Soret coefficients and fractions of E and EI_1 in the sample without I_2.

Amplitude as a function of $\mathbf{I_1}$ concentration and affinity between $\mathbf{I_1}$ and $\mathbf{E}$

$c_0^{EI_1}$ can be expressed in terms of total I_1, total E, and the dissociation constant between molecules I_1 and E that we denote as $K_D^{EI_1}$:

$$c_0^{I_1\,total} = c_0^{EI_1} + c_0^{I_1}$$

$$c_0^{E\,total} = c_0^{EI_1} + \square_0^E$$

$$K_D^{EI_1} = \frac{c_0^E c_0^{I_1}}{c_0^{EI_1}}$$

$$c_0^{EI_1} = \frac{c_0^{I_1\,total} + c_0^{E\,total} + K_D^{EI_1} - \sqrt{(c_0^{I_1\,total} + c_0^{E\,total} + K_D^{EI_1})^2 - 4c_0^{I_1\,total}c_0^{E\,total}}}{2}$$

Thus, $c_0^{EI_1}$ is a function of total I_1, total E, and $K_D^{EI_1}$:

$$c_0^{EI_1} = f\left(c_0^{\square_1\,total}, c_0^{E\,total}, K_D^{EI_1}\right) \qquad eq.(14)$$

c_0^E is also a function of total I_1, total E, and $K_D^{EI_1}$:

$$c_0^E = c_0^{E\,total} - c_0^{EI_1} = f\left(c_0^{I_1\,total}, c_0^{E\,total}, K_D^{EI_1}\right) \qquad eq.(15)$$

Finally, from $eq.(13) - (15)$:

$$Amplitude = f(c_0^{I_1\,total}, c_0^{E\,total}, K_D^{EI_1})$$

In our experimental assay, the total concentration of E is defined: it is the concentration of labeled NE that we add to plasma. Therefore, the $Amplitude$ of the obtained binding curves can differ only if the total concentration of I_1 – AAT in the plasma – or the affinity between I_1 and E – affinity between

AAT and NE – is different between the samples. In other words, $Amplitude$ reports on concentration of AAT and its affinity to NE in plasma samples.

Reference

1. Lippok, S. *et al. Anal. Chem.* **84,** 3523–30 (2012).

Supplementary Table 1

Measured values for FEV_1, concentration of AAT, and thermophoretic amplitude

	FEV_1 [%]	concentration AAT [nM]	thermophoretic amplitude
1	20.0	1070	39.62
2	32.0	1440	29.44
3	32.5	3820	35.68
4	34.0	4330	35.46
5	35.0	3170	33.00
6	39.0	1610	31.45
7	40.5	2340	32.46
8	42.0	1480	31.12
9	42.3	3360	32.09
10	45.0	1860	29.31
11	48.0	2710	34.51
12	50.0	2590	33.49
13	51.0	2230	30.83
14	54.0	4170	33.71
15	57.0	3990	31.68
16	60.0	2870	32.22
17	61.0	3790	31.19
18	69.2	2870	30.16
19	98.9	2270	30.11
20	100.7	2020	30.25
21	101.2	3270	28.37
22	110.0	3330	25.87
23	114.0	3250	30.93
24	115.6	2160	27.83
25	121.9	3300	29.87
26	130.1	4180	28.03

Supplementary Fig.1

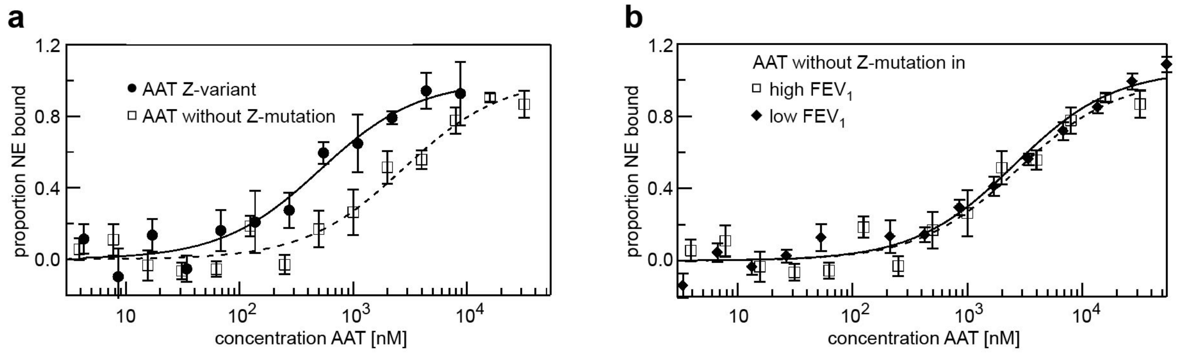

The AAT-variant with three stabilizing mutations (control) is less susceptible to plasma-dependent changes than the same variant with the Z-mutation.

(a) In the high FEV_1 (≥ 80%) plasma pool, the K_D between the stabilized Z-variant of AAT and NE ($K_{D(AAT/NE)}$ = 500 ± 100 nM) was approximately five times better than the K_D between the stabilized AAT without Z-mutation and NE ($K_{D(AAT/NE)}$ = 2760 ± 550 nM). Fitted binding curves and $K_D(EI_1)$ values (mean ± S.D.) were derived from global fitting of four measurements (three protein expressions) with Z-variant of AAT and three measurements (three protein expressions) with AAT without Z-mutation. (b) We compared the affinity between control AAT and NE in two pools of plasma from individuals with high FEV_1 (≥ 80%, n = 8) and low FEV_1 (≤ 50%, n = 12). There was no significant difference between high FEV_1 (≥ 80%) ($K_{D(AAT/NE)}$ = 2760 ± 550 nM) and low FEV_1 (≤ 50%) ($K_{D(AAT/NE)}$ = 2540 ± 300 nM). Fitted binding curves and $K_D(EI_1)$ values (mean ± S.D.) were derived from global fitting of three measurements (three protein expressions) in high FEV_1 plasma and four measurements (two protein expressions) in low FEV_1 plasma.

The measurements were performed in 7.5 % plasma and with 5 nM NE. Presented binding curves represent example measurements where each measurement point (mean ± S.D.) was derived from three technical replicates.
